# Ductal Carcinoma In Situ in Borderline Phyllodes Tumor: A Diagnostic and Treatment Dilemma

**DOI:** 10.7759/cureus.70012

**Published:** 2024-09-23

**Authors:** Sanghamitra Jena, Neetesh K Sinha, Bijan K Saha

**Affiliations:** 1 Surgical Oncology, Tata Main Hospital, Jamshedpur, IND

**Keywords:** borderline, breast carcinoma, ductal carcinoma in situ, phyllodes tumor, round block oncoplasty

## Abstract

The co-existence of breast carcinoma in the form of ductal carcinoma in situ (DCIS) in a case of Phyllodes tumor (PT) is extremely rare. We present a case of a pre-menopausal lady with a large breast lump diagnosed as benign PT on her initial biopsy. Wide local excision and breast conservation with round block oncoplasty were done. A post-operative diagnosis of borderline PT with sclerosing adenosis and high-grade DCIS were made. Adjuvant radiotherapy and hormonal therapy were given, and the patient had no recurrence after three years of follow-up.

DCIS in PT is very rare, and hence no standard protocol for treating such cases exists. So proper histopathological diagnosis, treatment with multidisciplinary involvement, and regular follow-up can help us conserve the breast and prevent recurrence in such cases.

## Introduction

Phyllodes tumors (PT) are rare tumors of the female breast, comprising 0.3-0.9% of all breast tumors [1-3]. These fibroepithelial neoplasms are characterized by biphasic proliferation of both stromal and epithelial components. Although both components of PT can undergo malignant transformation, it predominantly occurs in the stromal component and very rarely in the epithelial component, accounting for only 1-2% of all PT cases [3,4]. Epithelial malignant transformation can manifest as ductal carcinoma in situ (DCIS), infiltrating ductal carcinoma (IDC), lobular carcinoma, tubular carcinoma, or squamous cell carcinoma. To the best of our knowledge, 26 cases of co-existing breast carcinoma within PT have been reported in English literature globally, of which only 18 cases were of pure DCIS within benign PT, whereas one case was of both DCIS and IDC in a borderline PT of the breast [5-9]. In this report, we describe a rare case of a borderline malignant PT with high-grade DCIS of the breast. Due to the rarity of this condition and the lack of standard treatment protocols for such patients, we encountered dilemmas in both diagnosis and treatment. Our patient presented with a large breast lump, which was preoperatively diagnosed as benign PT. She was keen on preserving her breast, so she underwent breast conservation surgery using the round block oncoplasty technique. The unusual diagnosis of synchronous disease in the final histopathology posed a challenge in deciding further treatment. This case report highlights how a multidisciplinary approach, clear surgical margins, meticulous histopathological assessment, and appropriate adjuvant radiotherapy and hormonal therapy enabled us to avoid mastectomy while achieving good cosmetic and oncological outcomes for the patient.

## Case presentation

A 42-year-old woman presented with a right breast lump for six years. The lump had grown slowly, but the patient neglected it until it occupied almost the entire breast, prompting her to seek care at a local hospital. There, she was advised to undergo a mastectomy, but she came to the Department of Surgical Oncology hoping for breast conservation. There was no significant family history of breast cancer or any other previous breast problems.

On examination, a 10 x 10 cm multilobulated, firm, non-tender lump was palpable in the right breast, occupying almost all quadrants. The lump was free from the underlying chest wall. There was no right axillary lymphadenopathy. The contralateral breast and axilla were normal. The systemic examination did not reveal any significant abnormalities.

**Figure 1:**
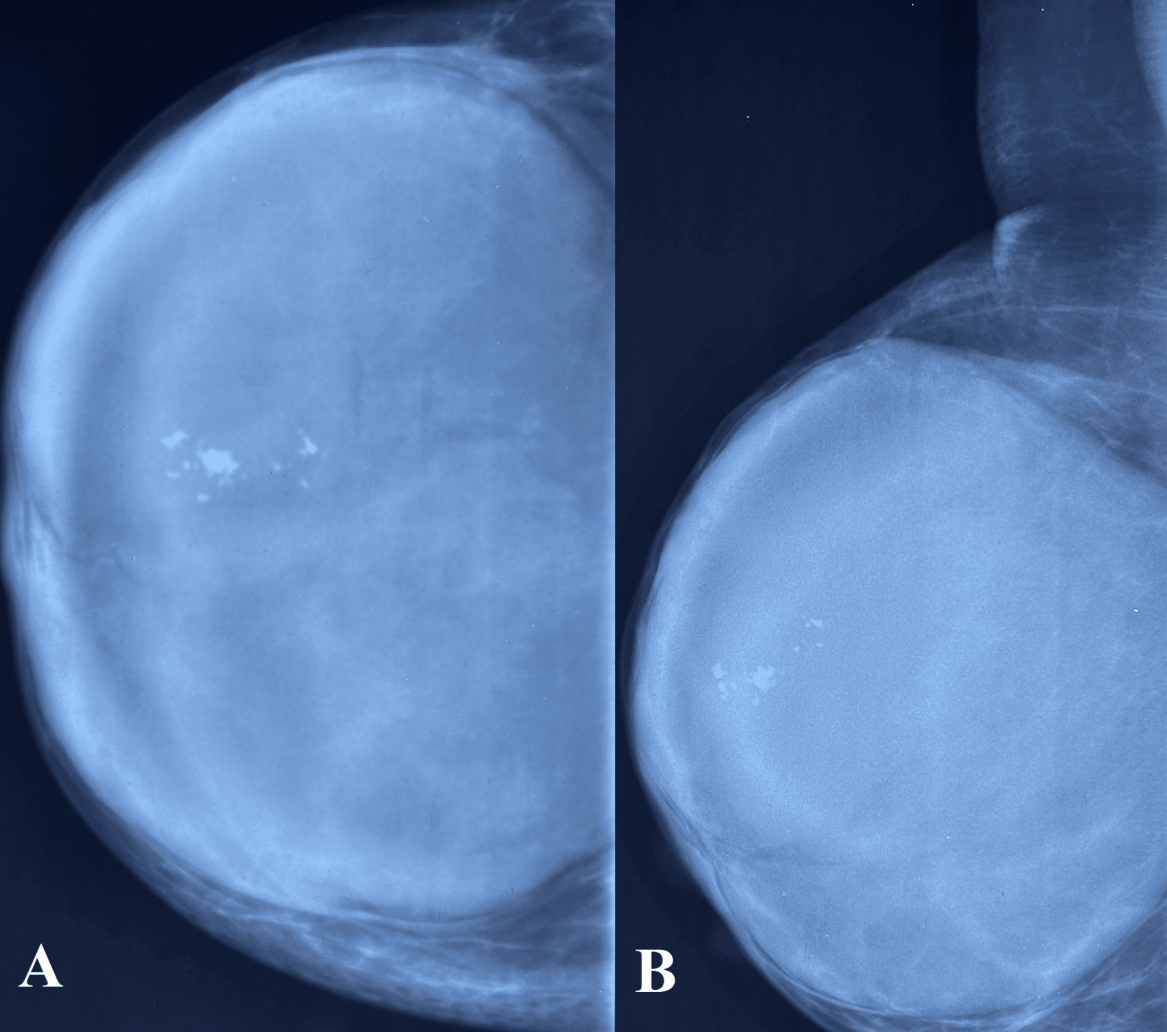
Mammography right breast. A) Craniocaudal view; B) Mediolateral oblique view Well-circumscribed hyperdense lesion occupying the entire area of the right breast, with coarse macrocalcifications in the upper outer quadrant.

Bilateral mammography revealed a large, well-defined, hyperdense lesion occupying the entire area of the right breast (Figure 1). The lesion had a well-circumscribed margin, with coarse macrocalcifications noted in the upper outer portion. The approximate measurements of the lesion were 12 x 5.2 x 12 cm. No significant lymph nodes were observed in the bilateral examination.

**Figure 2:**
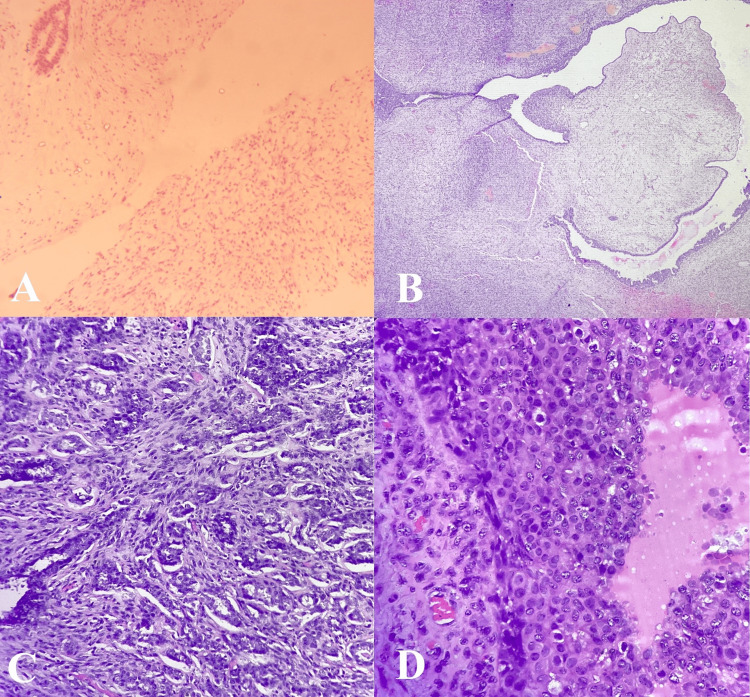
A) Core needle biopsy showing proliferation of spindle-shaped cells arranged haphazardly with myxoid appearance and few ducts lined by bilayered epithelial cells (20X H&E); B) Section showing cleft-like spaces surrounded by stromal overgrowth and few compressed ducts favoring borderline phyllodes tumor (20X H&E); C) Sclerosing adenosis (20X H&E); D) High-grade DCIS component with marked nuclear atypia and increased mitotic activity (40X H&E)

A core needle biopsy from the right breast lump showed the proliferation of uniform spindle-shaped cells arranged haphazardly, with myxoid appearance suggesting stromal overgrowth (Figure 2A). Few ducts were visible, lined by bilayered epithelial lining and focal epithelial hyperplasia. Nuclear atypia, mitosis, and necrosis were not identified. The impression was a fibroepithelial lesion, favoring fibroadenoma over PT.

**Figure 3:**
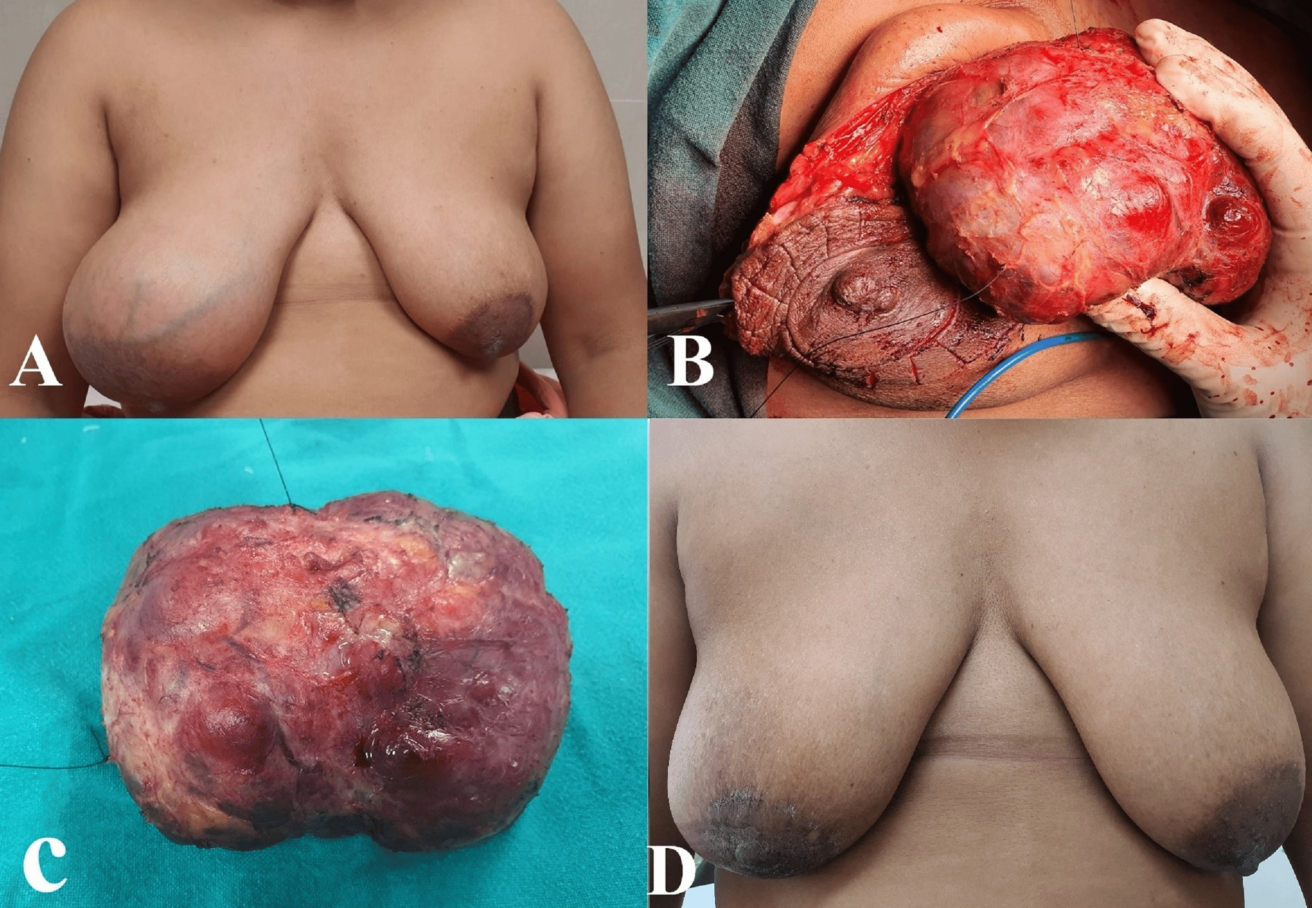
Round block oncoplasty A) Pre-operative picture showing gross asymmetry due to large mass in the right breast; B) Intra-operative picture showing excision of the tumor through the peri-areolar incision; C) Tumor after excision; D) Picture after three years of surgery

The case was discussed at the multidisciplinary tumor board, and surgery was planned. Wide local excision with a 1 cm margin and breast reconstruction using round block oncoplasty were performed (Figure 3). In the round block oncoplasty procedure, two concentric incisions were made around the areola, followed by de-epithelialization of the area between the incisions. The incision between the two concentric rings, near the tumor, was deepened, and the tumor was excised. The remaining breast tissue was brought together, and the defect was closed using a purse-string suture.

Macroscopically, the tumor dimension was 11 x 10 x 6 cm and weighed 433 grams. Serial cross-sections revealed a solid, whitish lesion with a flesh-fish-like appearance. Focal places showed cystic degeneration and congestion. Additionally, there was a calcified area measuring 3 x 1.5 cm.

Microscopic examination of the excised specimen showed a proliferative breast lesion comprising both mesenchymal and epithelial elements. The mesenchymal element showed stromal overgrowth, mild cellular atypia, hyalinization, and calcification. Mitotic count was 3-5/10 high power fields. At the periphery of the lesion, the mesenchymal element had a pushing as well as permeative border. However, no heterogeneous element of malignant nature was seen. The epithelial component showed proliferation, moderate atypia, and increased mitosis. Occasional papillary configuration and arcade of cells were present without any central necrosis of comedo type. No invasive epithelial component or features of metaplastic carcinoma were seen. All margins were negative for the tumor. On immunohistochemistry, the luminal cells were positive for estrogen receptor (ER). So, the final diagnosis of borderline PT with sclerosing adenosis and DCIS (high-grade amounting to approximately 10%) was made (Figures 2B-2D).

As per the decision of the multidisciplinary tumor board, the patient received adjuvant radiation (40 Grey in 15 fractions) and hormonal therapy (Tamoxifen 20 mg). The patient was reviewed after one month and then every three months thereafter. An excellent cosmetic outcome with bilateral symmetry was achieved. Annual mammography is being done, and after a follow-up of three years, she has no sign of clinical or radiological recurrence.

## Discussion

The WHO has categorized PTs into three groups based on their malignant potential, using various histological parameters. A benign PT typically exhibits less than five mitoses per 10 high-power fields (HPFs), mild stromal cellularity, well-defined tumor borders, minimal to mild atypia, and no stromal overgrowth. In contrast, the malignant group is marked by 10 or more mitosis per 10 HPFs, infiltrative margins, marked stromal cellularity and atypia, and stromal overgrowth [10]. The borderline PTs are characterized by five to nine mitotic figures per 10 HPFs, with histological characteristics lying between those of the other two categories. Benign PT comprises 60-70%, borderline PT 20-30%, and malignant PT 10% of all PT cases [11].

The coexistence of PTs with breast carcinoma is rare. These carcinomas may be found within the PT itself or as a separate lesion in the same or opposite breast [12,13]. Synchronous DCIS within a PT should be distinguished from carcinosarcoma, which carries a worse prognosis [14].

Given that the DCIS lesion is small compared to the PT itself, along with the lack of distinguishing clinical and radiological signs and the rarity of this coexistence, its diagnosis before surgery is challenging [11]. Most of the time, the diagnosis is confirmed only after the final histopathological examination and immunohistochemistry [13]. In our case, the diagnosis was missed in the initial biopsy and confirmed in the definitive surgical specimen.

Rare presentations of synchronous DCIS in PT can complicate post-operative treatment decisions and follow-up care. Surgical options for PT include breast conservation surgery and mastectomy, but axillary clearance is generally avoided since PTs rarely metastasize to lymph nodes [15]. According to the National Comprehensive Cancer Network (NCCN) guidelines, the management of DCIS involves breast conservation without lymph node surgery or mastectomy with sentinel lymph node biopsy [16]. In our case, since the surgical margins were clear and post-operative mammography showed no signs of residual disease, mastectomy was avoided. This is likely the first reported case of a borderline PT with high-grade DCIS successfully treated with breast conservation using the round block oncoplastic technique [17].

Adjuvant radiotherapy (RT) can be given to the breast post-excision of PT to prevent recurrence [18]. The RT is commonly indicated in malignant and borderline PT. Adjuvant RT is also required in cases of DCIS treated with breast-conserving surgeries [16]. So, adjuvant RT (40 Grey in 15 fractions) was given to this patient.

Adjuvant hormonal therapy can be used for PT patients with DCIS or IDC if they are ER or progesterone receptor positive [19]. In our patient, Tamoxifen 20 mg was started as she was strongly positive for ER. The patient is disease-free after a follow-up of three years.

## Conclusions

There is no existing guideline for the treatment of synchronous high-grade DCIS within borderline PT. Hence, clinicians, pathologists, and radiologists should be aware of this rare situation. Meticulous surgery, proper adjuvant radiation, and hormonal therapy can prevent the recurrence of the disease. Regular follow-up is also vital, as data regarding the prognosis and survival of this unusual lesion is limited. We managed to conserve the breast and were able to give a satisfactory oncological outcome despite the patient presenting with a large breast lump and the rarity of the disease.
